# Redetermination of dysprosium trinickel from single-crystal X-ray data

**DOI:** 10.1107/S1600536812043747

**Published:** 2012-10-27

**Authors:** Volodymyr Levytskyy, Volodymyr Babizhetskyy, Bohdan Kotur, Volodymyr Smetana

**Affiliations:** a Department of Inorganic Chemistry, Ivan Franko National University of Lviv, Kyryla & Mefodiya street 6, 79005 Lviv, Ukraine; b 344 Spedding Hall, Ames Laboratory, Ames, IA 50011-3020, USA

## Abstract

The crystal structure of the title compound, DyNi_3_, was redetermined from single-crystal X-ray diffraction data. In comparison with previous studies based on powder X-ray diffraction data [Lemaire & Paccard (1969 ▶). *Bull. Soc. Fr. Minéral. Cristallogr.* **92**, 9–16; Tsai *et al.* (1974 ▶). *J. Appl. Phys.* **45**, 3582–3586], the present redetermination revealed refined coordinates and anisotropic displacement parameters for all atoms. The crystal structure of DyNi_3_ adopts the PuNi_3_ structure type and can be derived from the CaCu_5_ structure type as an inter­growth structure. The asymmetric unit contains two Dy sites (site symmetries 3*m* and -3) and three Ni sites (*m*, 3*m* and -3*m*). The two different coordination polyhedra of Dy are a Frank–Kasper polyhedron formed by four Dy and 12 Ni atoms and a pseudo-Frank–Kasper polyhedron formed by two Dy and 18 Ni atoms. The three different coordination polyhedra of Ni are Frank–Kasper icosa­hedra formed by five Dy and seven Ni atoms, three Dy and nine Ni atoms, and six Dy and six Ni atoms.

## Related literature

For the PuNi_3_ structure type, see: Cromer & Olsen (1959 ▶). For previous powder diffraction studies of the title compoud, see: Paccard & Pauthenet (1967 ▶); Lemaire & Paccard (1969 ▶); Virkar & Raman (1969 ▶); Buschow & van der Goot (1970 ▶); Yakinthos & Paccard (1972 ▶); Tsai *et al.* (1974 ▶). For related compounds, see: Virkar & Raman (1969 ▶); Buschow & van der Goot (1970 ▶); Levytskyy *et al.* (2012 ▶). For the CaCu_5_ structure type, see: Haucke (1940 ▶); Nowotny (1942 ▶). For the MgCu_2_ structure type, see: Friauf (1927 ▶); Ohba *et al.* (1984 ▶). For inter­growth structures, see: Parthé *et al.* (1985 ▶); Grin (1992 ▶).

## Experimental

### 

#### Crystal data

DyNi_3_
*M* *_r_* = 338.63
Trigonal, 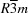

*a* = 4.966 (2) Å
*c* = 24.37 (1) Å
*V* = 520.5 (4) Å^3^
*Z* = 9
Mo *K*α radiation
μ = 55.52 mm^−1^
*T* = 293 K
0.13 × 0.08 × 0.06 mm

#### Data collection

Stoe IPDS II diffractometer
Absorption correction: multi-scan (*PLATON*, Spek, 2009 ▶) *T*_min_ = 0.071, *T*_max_ = 0.182
1516 measured reflections
197 independent reflections
163 reflections with *I* > 2σ(*I*)
*R*_int_ = 0.058

#### Refinement

*R*[*F* ^2^ > 2σ(*F* ^2^)] = 0.022
*wR*(*F* ^2^) = 0.043
*S* = 1.01
197 reflections
17 parameters
Δρ_max_ = 2.77 e Å^−3^
Δρ_min_ = −1.33 e Å^−3^

### 

Data collection: *X-AREA* (Stoe & Cie, 2009 ▶); cell refinement: *X-AREA*; data reduction: *X-AREA*; program(s) used to solve structure: *SIR2011* (Burla *et al.*, 2012 ▶); program(s) used to refine structure: *SHELXL97* (Sheldrick, 2008 ▶) and *WinGX* (Farrugia, 1999 ▶); molecular graphics: *DIAMOND* (Brandenburg, 2006 ▶); software used to prepare material for publication: *publCIF* (Westrip, 2010 ▶).

## Supplementary Material

Crystal structure: contains datablock(s) I, global. DOI: 10.1107/S1600536812043747/wm2688sup1.cif

Structure factors: contains datablock(s) I. DOI: 10.1107/S1600536812043747/wm2688Isup2.hkl

Additional supplementary materials:  crystallographic information; 3D view; checkCIF report

## Figures and Tables

**Fig. 1.**
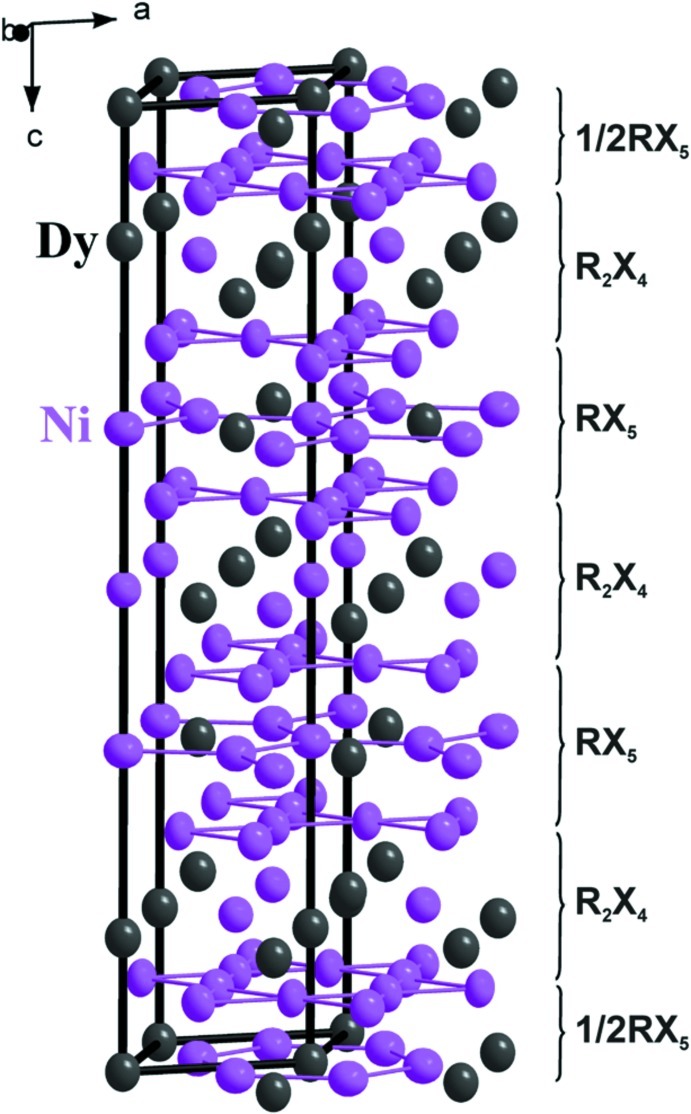
Perspective view of the crystal structure of DyNi3. The unit cell and the blocks of RX5 and R2X4 are emphasized. Atoms are represented by their anisotropic displacement ellipsoids at the 99.9% probability level

**Fig. 2.**
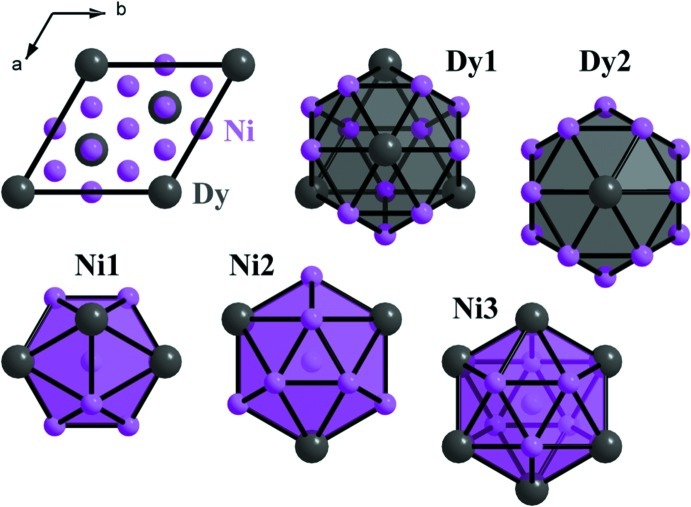
The ab projection of the unit cell and coordination polyhedra for all types of atoms in the DyNi3 structure

## supplementary crystallographic information

### Comment

The existence of the intermetallic phase with composition DyNi_3_ has been long known before. The first structure report (Paccard & Pauthenet, 1967) of the title compound revealed isotypism with the PuNi_3_ structure type (Cromer & Olsen, 1959). Lattice parameters were determined from X-ray powder diffraction data without specifying atomic coordinates (Paccard & Pauthenet, 1967; Lemaire & Paccard, 1969; Virkar & Raman, 1969; Buschow & van der Goot, 1970; Tsai *et al.*, 1974). Yakinthos & Paccard (1972) reported crystal structure data for *R*Ni_3_ compounds (*R* = Pr, Nd, Tb, Dy, Tm) from powder neutron diffraction data.

The present work contains the results of the full single-crystal X-ray determination of DyNi_3_, including refinement of the atomic coordinates and the temperature factors for all atoms. These results confirm the belonging to the PuNi_3_ structure type in space group *R*3*m*. A view of the crystal structure of DyNi_3_ is shown in Fig. 1. As has been noted previously (Yakinthos & Paccard, 1972), the crystal structure of DyNi_3_ can be derived from the CaCu_5_ structure type (Haucke, 1940; Nowotny, 1942). It consists of stacks of *RX*_5_ blocks (CaCu_5_-type) and *R*_2_*X*_4_ blocks (MgCu_2_-type (Friauf, 1927; Ohba *et al.*, 1984)). Both types have the same Kagome net of Ni atoms that allows a combination of both structural motifs along the 3-fold inversion axis. As a result, it can be considered as an intergrowth structure: *R*_2_*X*_4_ + *RX*_5_ = 3*RX*_3_ (Parthé *et al.*, 1985; Grin, 1992).

In Fig. 2 the projection of the unit cell on the *ab* plane and the resulting coordination polyhedra for all atom types are shown. The coordination number for the Dy1 atom (Wyckoff site 6*c*, site symmetry 3*m*). The coordination polyhedron for this atom is a Frank-Kasper polyhedron formed by 4 Dy and 12 Ni atoms. The coordination number for the Dy2 atom (Wyckoff site 3*a*, site symmetry 3*m*) is 20. The coordination polyhedron of Dy2 is a pseudo-Frank-Kasper polyhedron formed by 2 Dy and 18 Ni atoms. Although the site symmetries for all Ni atoms are different, the coordination number for all Ni atoms is 12, with Frank-Kasper icosahedra as coordination polyhedra. The Ni1 atom (Wyckoff site 18*h*, site symmetry *.m*) is surrounded by 5 Dy atoms and 7 Ni atoms. The Ni2 atom (Wyckoff site 6*c*, site symmetry 3*m*) is surrounded by 3 Dy atoms and 9 Ni atoms. The Ni3 atom (Wyckoff site 3*b*, site symmetry 3*m*) is surrounded by 6 Dy atoms and 6 Ni atoms.

The interatomic distances in DyNi_3_ are similar than those in Di_2_Ni_7_ (Levytskyy *et al.*, 2012).

### Experimental

The sample was prepared of the powdered commercially available pure elements: sublimed bulk pieces of dysprosium metal with a claimed purity of 99.99 at.% (Alfa Aesar, Johnson Matthey) and electrolytic nickel (99.99% pure) piece (Aldrich). A mixture of the powders was compacted in stainless steel dies. The pellet was arc-melted under an argon atmosphere on a water-cooled copper hearth. The alloy button (~1 g) was turned over and remolten three times to improve homogeneity. Subsequently, the sample was annealed in an evacuated silica tube under an argon atmosphere for four weeks at 1070 K. Shiny grey irregular-shaped crystals were isolated mechanically with a help of microscope by crushing the sample.

### Refinement

The atomic positions found from the direct methods structure solution were in good agreement with those from the PuNi_3_ structure type and were used as starting parameters for the structure refinement. The highest Fourier difference peak of 2.77 e Å^-3^ is at (0 0 0.1019) and 0.91 Å away from the Dy1 atom. The deepest hole of -1.33 e Å^-3^ is at (0.8263 0.9132 0.0194) and 0.88 Å away from the Dy2 atom.

### Crystal data

**Table:**

DyNi_3_	*D*_x_ = 9.723 Mg m^−^^3^
*M**_r_* = 338.63	Mo *K*α radiation, λ = 0.71069 Å
Trigonal, *R*3*m*	Cell parameters from 1064 reflections
Hall symbol: -R 3 2"	θ = 0.8–28.4°
*a* = 4.966 (2) Å	µ = 55.52 mm^−^^1^
*c* = 24.37 (1) Å	*T* = 293 K
*V* = 520.5 (4) Å^3^	Irregular, grey
*Z* = 9	0.13 × 0.08 × 0.06 mm
*F*(000) = 1350	

### Data collection

**Table:**

Stoe IPDS II diffractometer	197 independent reflections
Radiation source: fine-focus sealed tube	163 reflections with *I* > 2σ(*I*)
Graphite monochromator	*R*_int_ = 0.058
ω scans	θ_max_ = 28.4°, θ_min_ = 2.5°
Absorption correction: multi-scan (*PLATON*, Spek, 2009)	*h* = −6→6
*T*_min_ = 0.071, *T*_max_ = 0.182	*k* = −6→6
1516 measured reflections	*l* = −32→30

### Refinement

**Table:**

Refinement on *F*^2^	0 restraints
Least-squares matrix: full	Primary atom site location: structure-invariant direct methods
*R*[*F*^2^ > 2σ(*F*^2^)] = 0.022	Secondary atom site location: difference Fourier map
*wR*(*F*^2^) = 0.043	*w* = 1/[σ^2^(*F*_o_^2^) + (0.0207*P*)^2^] where *P* = (*F*_o_^2^ + 2*F*_c_^2^)/3
*S* = 1.01	(Δ/σ)_max_ < 0.001
197 reflections	Δρ_max_ = 2.77 e Å^−^^3^
17 parameters	Δρ_min_ = −1.33 e Å^−^^3^

### Special details

**Table:**

Geometry. All e.s.d.'s (except the e.s.d. in the dihedral angle between two l.s. planes) are estimated using the full covariance matrix. The cell e.s.d.'s are taken into account individually in the estimation of e.s.d.'s in distances, angles and torsion angles; correlations between e.s.d.'s in cell parameters are only used when they are defined by crystal symmetry. An approximate (isotropic) treatment of cell e.s.d.'s is used for estimating e.s.d.'s involving l.s. planes.
Refinement. Refinement of *F*^2^ against ALL reflections. The weighted *R*-factor *wR* and goodness of fit *S* are based on *F*^2^, conventional *R*-factors *R* are based on *F*, with *F* set to zero for negative *F*^2^. The threshold expression of *F*^2^ > σ(*F*^2^) is used only for calculating *R*-factors(gt) *etc*. and is not relevant to the choice of reflections for refinement. *R*-factors based on *F*^2^ are statistically about twice as large as those based on *F*, and *R*- factors based on ALL data will be even larger.

### Fractional atomic coordinates and isotropic or equivalent isotropic displacement parameters (Å^2^)

**Table:**

	*x*	*y*	*z*	*U*_iso_*/*U*_eq_	
Ni1	0.50038 (13)	0.49962 (13)	0.08188 (5)	0.0116 (3)	
Dy1	0.0000	0.0000	0.13920 (4)	0.0135 (2)	
Ni2	0.0000	0.0000	0.33306 (9)	0.0143 (5)	
Ni3	0.0000	0.0000	0.5000	0.0118 (7)	
Dy2	0.0000	0.0000	0.0000	0.0128 (3)	

### Atomic displacement parameters (Å^2^)

**Table:**

	*U*^11^	*U*^22^	*U*^33^	*U*^12^	*U*^13^	*U*^23^
Ni1	0.0112 (5)	0.0112 (5)	0.0141 (7)	0.0069 (6)	0.0003 (3)	−0.0003 (3)
Dy1	0.0125 (3)	0.0125 (3)	0.0157 (5)	0.00623 (15)	0.000	0.000
Ni2	0.0153 (7)	0.0153 (7)	0.0123 (12)	0.0077 (4)	0.000	0.000
Ni3	0.0115 (10)	0.0115 (10)	0.0124 (17)	0.0057 (5)	0.000	0.000
Dy2	0.0117 (4)	0.0117 (4)	0.0151 (6)	0.00584 (19)	0.000	0.000

### Geometric parameters (Å, º)

**Table:**

Ni1—Ni2^i^	2.450 (2)	Ni2—Dy2^xviii^	2.8671 (12)
Ni1—Ni2^ii^	2.464 (2)	Ni2—Dy2^xv^	2.8671 (12)
Ni1—Ni1^iii^	2.477 (2)	Ni2—Ni2^xix^	2.8671 (12)
Ni1—Ni1^iv^	2.477 (2)	Ni2—Ni2^xx^	2.8671 (12)
Ni1—Ni1^v^	2.489 (2)	Ni2—Dy2^xxi^	2.8672 (12)
Ni1—Ni1^vi^	2.489 (2)	Ni2—Ni2^xxii^	2.8672 (12)
Ni1—Ni3^ii^	2.5166 (14)	Ni3—Ni1^xx^	2.5166 (14)
Ni1—Dy1^vii^	2.8489 (11)	Ni3—Ni1^xv^	2.5166 (14)
Ni1—Dy1	2.8489 (11)	Ni3—Ni1^xxiii^	2.5166 (14)
Ni1—Dy1^i^	3.0869 (18)	Ni3—Ni1^xvii^	2.5166 (14)
Ni1—Dy2	3.1855 (12)	Ni3—Ni1^xxiv^	2.5166 (14)
Ni1—Dy2^vii^	3.1855 (12)	Ni3—Ni1^xvi^	2.5166 (14)
Dy1—Ni1^viii^	2.8489 (11)	Ni3—Dy1^xx^	2.9442 (11)
Dy1—Ni1^ix^	2.8489 (11)	Ni3—Dy1^xv^	2.9442 (11)
Dy1—Ni1^x^	2.8489 (11)	Ni3—Dy1^xix^	2.9442 (11)
Dy1—Ni1^vi^	2.8489 (11)	Ni3—Dy1^xviii^	2.9442 (11)
Dy1—Ni1^iv^	2.8489 (11)	Ni3—Dy1^xxii^	2.9442 (11)
Dy1—Ni3^ii^	2.9442 (11)	Ni3—Dy1^xxi^	2.9442 (11)
Dy1—Ni3^xi^	2.9442 (11)	Dy2—Ni2^i^	2.8671 (12)
Dy1—Ni3^xii^	2.9442 (11)	Dy2—Ni2^xxv^	2.8671 (12)
Dy1—Ni1^i^	3.0869 (18)	Dy2—Ni2^ii^	2.8671 (12)
Dy1—Ni1^xiii^	3.0869 (18)	Dy2—Ni2^xi^	2.8671 (12)
Dy1—Ni1^xiv^	3.0869 (18)	Dy2—Ni2^xxvi^	2.8672 (12)
Ni2—Ni1^i^	2.450 (2)	Dy2—Ni2^xii^	2.8672 (12)
Ni2—Ni1^xiv^	2.450 (2)	Dy2—Ni1^xxvii^	3.1855 (12)
Ni2—Ni1^xiii^	2.450 (2)	Dy2—Ni1^vi^	3.1855 (12)
Ni2—Ni1^xv^	2.464 (2)	Dy2—Ni1^iv^	3.1855 (12)
Ni2—Ni1^xvi^	2.464 (2)	Dy2—Ni1^xxviii^	3.1855 (12)
Ni2—Ni1^xvii^	2.464 (2)	Dy2—Ni1^viii^	3.1855 (12)
			
Ni2^i^—Ni1—Ni2^ii^	71.39 (4)	Ni1^xvii^—Ni2—Ni2^xix^	54.07 (7)
Ni2^i^—Ni1—Ni1^iii^	59.63 (4)	Dy2^xviii^—Ni2—Ni2^xix^	179.60 (14)
Ni2^ii^—Ni1—Ni1^iii^	120.32 (4)	Dy2^xv^—Ni2—Ni2^xix^	60.0
Ni2^i^—Ni1—Ni1^iv^	59.63 (4)	Ni1^i^—Ni2—Ni2^xx^	107.20 (9)
Ni2^ii^—Ni1—Ni1^iv^	120.32 (4)	Ni1^xiv^—Ni2—Ni2^xx^	107.20 (9)
Ni1^iii^—Ni1—Ni1^iv^	60.0	Ni1^xiii^—Ni2—Ni2^xx^	54.54 (7)
Ni2^i^—Ni1—Ni1^v^	120.37 (4)	Ni1^xv^—Ni2—Ni2^xx^	106.72 (9)
Ni2^ii^—Ni1—Ni1^v^	59.68 (4)	Ni1^xvi^—Ni2—Ni2^xx^	54.07 (7)
Ni1^iii^—Ni1—Ni1^v^	120.0	Ni1^xvii^—Ni2—Ni2^xx^	106.72 (9)
Ni1^iv^—Ni1—Ni1^v^	180.0	Dy2^xviii^—Ni2—Ni2^xx^	60.0
Ni2^i^—Ni1—Ni1^vi^	120.37 (4)	Dy2^xv^—Ni2—Ni2^xx^	179.60 (14)
Ni2^ii^—Ni1—Ni1^vi^	59.68 (4)	Ni2^xix^—Ni2—Ni2^xx^	119.999 (2)
Ni1^iii^—Ni1—Ni1^vi^	180.00 (5)	Ni1^i^—Ni2—Dy2^xxi^	125.86 (8)
Ni1^iv^—Ni1—Ni1^vi^	120.0	Ni1^xiv^—Ni2—Dy2^xxi^	73.14 (3)
Ni1^v^—Ni1—Ni1^vi^	60.0	Ni1^xiii^—Ni2—Dy2^xxi^	73.14 (3)
Ni2^i^—Ni1—Ni3^ii^	179.09 (6)	Ni1^xv^—Ni2—Dy2^xxi^	125.53 (8)
Ni2^ii^—Ni1—Ni3^ii^	109.52 (6)	Ni1^xvi^—Ni2—Dy2^xxi^	72.94 (3)
Ni1^iii^—Ni1—Ni3^ii^	119.63 (2)	Ni1^xvii^—Ni2—Dy2^xxi^	72.94 (3)
Ni1^iv^—Ni1—Ni3^ii^	119.63 (2)	Dy2^xviii^—Ni2—Dy2^xxi^	119.999 (1)
Ni1^v^—Ni1—Ni3^ii^	60.37 (2)	Dy2^xv^—Ni2—Dy2^xxi^	119.999 (1)
Ni1^vi^—Ni1—Ni3^ii^	60.37 (2)	Ni2^xix^—Ni2—Dy2^xxi^	60.0
Ni2^i^—Ni1—Dy1^vii^	113.50 (3)	Ni2^xx^—Ni2—Dy2^xxi^	60.0
Ni2^ii^—Ni1—Dy1^vii^	113.43 (3)	Ni1^i^—Ni2—Ni2^xxii^	54.54 (7)
Ni1^iii^—Ni1—Dy1^vii^	64.23 (2)	Ni1^xiv^—Ni2—Ni2^xxii^	107.20 (9)
Ni1^iv^—Ni1—Dy1^vii^	115.90 (2)	Ni1^xiii^—Ni2—Ni2^xxii^	107.20 (9)
Ni1^v^—Ni1—Dy1^vii^	64.10 (2)	Ni1^xv^—Ni2—Ni2^xxii^	54.07 (7)
Ni1^vi^—Ni1—Dy1^vii^	115.77 (2)	Ni1^xvi^—Ni2—Ni2^xxii^	106.72 (9)
Ni3^ii^—Ni1—Dy1^vii^	66.22 (3)	Ni1^xvii^—Ni2—Ni2^xxii^	106.72 (9)
Ni2^i^—Ni1—Dy1	113.50 (3)	Dy2^xviii^—Ni2—Ni2^xxii^	60.0
Ni2^ii^—Ni1—Dy1	113.43 (3)	Dy2^xv^—Ni2—Ni2^xxii^	60.0
Ni1^iii^—Ni1—Dy1	115.90 (2)	Ni2^xix^—Ni2—Ni2^xxii^	119.997 (2)
Ni1^iv^—Ni1—Dy1	64.23 (2)	Ni2^xx^—Ni2—Ni2^xxii^	119.997 (2)
Ni1^v^—Ni1—Dy1	115.77 (2)	Dy2^xxi^—Ni2—Ni2^xxii^	179.60 (14)
Ni1^vi^—Ni1—Dy1	64.10 (2)	Ni1^xx^—Ni3—Ni1^xv^	180.00 (3)
Ni3^ii^—Ni1—Dy1	66.22 (3)	Ni1^xx^—Ni3—Ni1^xxiii^	59.27 (5)
Dy1^vii^—Ni1—Dy1	121.28 (6)	Ni1^xv^—Ni3—Ni1^xxiii^	120.73 (5)
Ni2^i^—Ni1—Dy1^i^	116.67 (6)	Ni1^xx^—Ni3—Ni1^xvii^	120.73 (5)
Ni2^ii^—Ni1—Dy1^i^	171.94 (6)	Ni1^xv^—Ni3—Ni1^xvii^	59.27 (5)
Ni1^iii^—Ni1—Dy1^i^	66.34 (2)	Ni1^xxiii^—Ni3—Ni1^xvii^	180.0
Ni1^iv^—Ni1—Dy1^i^	66.34 (2)	Ni1^xx^—Ni3—Ni1^xxiv^	59.27 (5)
Ni1^v^—Ni1—Dy1^i^	113.66 (2)	Ni1^xv^—Ni3—Ni1^xxiv^	120.73 (5)
Ni1^vi^—Ni1—Dy1^i^	113.66 (2)	Ni1^xxiii^—Ni3—Ni1^xxiv^	59.27 (5)
Ni3^ii^—Ni1—Dy1^i^	62.42 (4)	Ni1^xvii^—Ni3—Ni1^xxiv^	120.73 (5)
Dy1^vii^—Ni1—Dy1^i^	64.28 (3)	Ni1^xx^—Ni3—Ni1^xvi^	120.73 (5)
Dy1—Ni1—Dy1^i^	64.28 (3)	Ni1^xv^—Ni3—Ni1^xvi^	59.27 (5)
Ni2^i^—Ni1—Dy2	59.47 (3)	Ni1^xxiii^—Ni3—Ni1^xvi^	120.73 (5)
Ni2^ii^—Ni1—Dy2	59.37 (3)	Ni1^xvii^—Ni3—Ni1^xvi^	59.27 (5)
Ni1^iii^—Ni1—Dy2	112.99 (2)	Ni1^xxiv^—Ni3—Ni1^xvi^	180.0
Ni1^iv^—Ni1—Dy2	67.12 (2)	Ni1^xx^—Ni3—Dy1^xx^	62.314 (16)
Ni1^v^—Ni1—Dy2	112.88 (2)	Ni1^xv^—Ni3—Dy1^xx^	117.686 (16)
Ni1^vi^—Ni1—Dy2	67.01 (2)	Ni1^xxiii^—Ni3—Dy1^xx^	62.314 (16)
Ni3^ii^—Ni1—Dy2	120.91 (3)	Ni1^xvii^—Ni3—Dy1^xx^	117.686 (16)
Dy1^vii^—Ni1—Dy2	170.57 (4)	Ni1^xxiv^—Ni3—Dy1^xx^	111.68 (4)
Dy1—Ni1—Dy2	68.15 (3)	Ni1^xvi^—Ni3—Dy1^xx^	68.32 (4)
Dy1^i^—Ni1—Dy2	123.75 (3)	Ni1^xx^—Ni3—Dy1^xv^	117.686 (16)
Ni2^i^—Ni1—Dy2^vii^	59.47 (3)	Ni1^xv^—Ni3—Dy1^xv^	62.314 (16)
Ni2^ii^—Ni1—Dy2^vii^	59.37 (3)	Ni1^xxiii^—Ni3—Dy1^xv^	117.686 (16)
Ni1^iii^—Ni1—Dy2^vii^	67.12 (2)	Ni1^xvii^—Ni3—Dy1^xv^	62.314 (16)
Ni1^iv^—Ni1—Dy2^vii^	112.99 (2)	Ni1^xxiv^—Ni3—Dy1^xv^	68.32 (4)
Ni1^v^—Ni1—Dy2^vii^	67.01 (2)	Ni1^xvi^—Ni3—Dy1^xv^	111.68 (4)
Ni1^vi^—Ni1—Dy2^vii^	112.88 (2)	Dy1^xx^—Ni3—Dy1^xv^	180.0
Ni3^ii^—Ni1—Dy2^vii^	120.91 (3)	Ni1^xx^—Ni3—Dy1^xix^	62.314 (16)
Dy1^vii^—Ni1—Dy2^vii^	68.15 (3)	Ni1^xv^—Ni3—Dy1^xix^	117.686 (16)
Dy1—Ni1—Dy2^vii^	170.57 (4)	Ni1^xxiii^—Ni3—Dy1^xix^	111.68 (4)
Dy1^i^—Ni1—Dy2^vii^	123.75 (3)	Ni1^xvii^—Ni3—Dy1^xix^	68.32 (4)
Dy2—Ni1—Dy2^vii^	102.42 (5)	Ni1^xxiv^—Ni3—Dy1^xix^	62.314 (16)
Ni1^viii^—Dy1—Ni1^ix^	51.80 (5)	Ni1^xvi^—Ni3—Dy1^xix^	117.686 (16)
Ni1^viii^—Dy1—Ni1^x^	51.54 (5)	Dy1^xx^—Ni3—Dy1^xix^	114.993 (13)
Ni1^ix^—Dy1—Ni1^x^	98.01 (4)	Dy1^xv^—Ni3—Dy1^xix^	65.007 (13)
Ni1^viii^—Dy1—Ni1	121.28 (6)	Ni1^xx^—Ni3—Dy1^xviii^	117.686 (16)
Ni1^ix^—Dy1—Ni1	98.01 (4)	Ni1^xv^—Ni3—Dy1^xviii^	62.314 (16)
Ni1^x^—Dy1—Ni1	98.01 (4)	Ni1^xxiii^—Ni3—Dy1^xviii^	68.32 (4)
Ni1^viii^—Dy1—Ni1^vi^	98.01 (4)	Ni1^xvii^—Ni3—Dy1^xviii^	111.68 (4)
Ni1^ix^—Dy1—Ni1^vi^	51.54 (5)	Ni1^xxiv^—Ni3—Dy1^xviii^	117.686 (16)
Ni1^x^—Dy1—Ni1^vi^	121.28 (6)	Ni1^xvi^—Ni3—Dy1^xviii^	62.314 (16)
Ni1—Dy1—Ni1^vi^	51.80 (5)	Dy1^xx^—Ni3—Dy1^xviii^	65.007 (13)
Ni1^viii^—Dy1—Ni1^iv^	98.01 (4)	Dy1^xv^—Ni3—Dy1^xviii^	114.993 (13)
Ni1^ix^—Dy1—Ni1^iv^	121.28 (6)	Dy1^xix^—Ni3—Dy1^xviii^	180.00 (3)
Ni1^x^—Dy1—Ni1^iv^	51.80 (5)	Ni1^xx^—Ni3—Dy1^xxii^	111.68 (4)
Ni1—Dy1—Ni1^iv^	51.54 (5)	Ni1^xv^—Ni3—Dy1^xxii^	68.32 (4)
Ni1^vi^—Dy1—Ni1^iv^	98.01 (4)	Ni1^xxiii^—Ni3—Dy1^xxii^	62.314 (16)
Ni1^viii^—Dy1—Ni3^ii^	147.89 (3)	Ni1^xvii^—Ni3—Dy1^xxii^	117.686 (16)
Ni1^ix^—Dy1—Ni3^ii^	96.33 (2)	Ni1^xxiv^—Ni3—Dy1^xxii^	62.314 (16)
Ni1^x^—Dy1—Ni3^ii^	147.89 (3)	Ni1^xvi^—Ni3—Dy1^xxii^	117.686 (16)
Ni1—Dy1—Ni3^ii^	51.46 (3)	Dy1^xx^—Ni3—Dy1^xxii^	114.992 (13)
Ni1^vi^—Dy1—Ni3^ii^	51.46 (3)	Dy1^xv^—Ni3—Dy1^xxii^	65.008 (13)
Ni1^iv^—Dy1—Ni3^ii^	96.33 (2)	Dy1^xix^—Ni3—Dy1^xxii^	114.992 (13)
Ni1^viii^—Dy1—Ni3^xi^	51.46 (3)	Dy1^xviii^—Ni3—Dy1^xxii^	65.008 (13)
Ni1^ix^—Dy1—Ni3^xi^	51.46 (3)	Ni1^xx^—Ni3—Dy1^xxi^	68.32 (4)
Ni1^x^—Dy1—Ni3^xi^	96.33 (2)	Ni1^xv^—Ni3—Dy1^xxi^	111.68 (4)
Ni1—Dy1—Ni3^xi^	147.89 (3)	Ni1^xxiii^—Ni3—Dy1^xxi^	117.686 (16)
Ni1^vi^—Dy1—Ni3^xi^	96.33 (2)	Ni1^xvii^—Ni3—Dy1^xxi^	62.314 (16)
Ni1^iv^—Dy1—Ni3^xi^	147.89 (3)	Ni1^xxiv^—Ni3—Dy1^xxi^	117.686 (16)
Ni3^ii^—Dy1—Ni3^xi^	114.993 (13)	Ni1^xvi^—Ni3—Dy1^xxi^	62.314 (16)
Ni1^viii^—Dy1—Ni3^xii^	96.33 (2)	Dy1^xx^—Ni3—Dy1^xxi^	65.008 (13)
Ni1^ix^—Dy1—Ni3^xii^	147.89 (3)	Dy1^xv^—Ni3—Dy1^xxi^	114.992 (13)
Ni1^x^—Dy1—Ni3^xii^	51.46 (3)	Dy1^xix^—Ni3—Dy1^xxi^	65.008 (13)
Ni1—Dy1—Ni3^xii^	96.33 (2)	Dy1^xviii^—Ni3—Dy1^xxi^	114.992 (13)
Ni1^vi^—Dy1—Ni3^xii^	147.89 (3)	Dy1^xxii^—Ni3—Dy1^xxi^	180.00 (3)
Ni1^iv^—Dy1—Ni3^xii^	51.46 (3)	Ni2^i^—Dy2—Ni2^xxv^	120.0
Ni3^ii^—Dy1—Ni3^xii^	114.992 (13)	Ni2^i^—Dy2—Ni2^ii^	60.0
Ni3^xi^—Dy1—Ni3^xii^	114.992 (13)	Ni2^xxv^—Dy2—Ni2^ii^	180.0
Ni1^viii^—Dy1—Ni1^i^	115.72 (3)	Ni2^i^—Dy2—Ni2^xi^	180.0
Ni1^ix^—Dy1—Ni1^i^	141.67 (2)	Ni2^xxv^—Dy2—Ni2^xi^	60.0
Ni1^x^—Dy1—Ni1^i^	94.88 (4)	Ni2^ii^—Dy2—Ni2^xi^	120.0
Ni1—Dy1—Ni1^i^	115.72 (3)	Ni2^i^—Dy2—Ni2^xxvi^	120.0
Ni1^vi^—Dy1—Ni1^i^	141.67 (2)	Ni2^xxv^—Dy2—Ni2^xxvi^	120.0
Ni1^iv^—Dy1—Ni1^i^	94.88 (4)	Ni2^ii^—Dy2—Ni2^xxvi^	60.0
Ni3^ii^—Dy1—Ni1^i^	91.38 (3)	Ni2^xi^—Dy2—Ni2^xxvi^	60.0
Ni3^xi^—Dy1—Ni1^i^	91.38 (3)	Ni2^i^—Dy2—Ni2^xii^	60.0
Ni3^xii^—Dy1—Ni1^i^	49.26 (2)	Ni2^xxv^—Dy2—Ni2^xii^	60.0
Ni1^viii^—Dy1—Ni1^xiii^	141.67 (2)	Ni2^ii^—Dy2—Ni2^xii^	120.0
Ni1^ix^—Dy1—Ni1^xiii^	115.72 (3)	Ni2^xi^—Dy2—Ni2^xii^	120.0
Ni1^x^—Dy1—Ni1^xiii^	141.67 (2)	Ni2^xxvi^—Dy2—Ni2^xii^	180.0
Ni1—Dy1—Ni1^xiii^	94.88 (4)	Ni2^i^—Dy2—Ni1	47.39 (4)
Ni1^vi^—Dy1—Ni1^xiii^	94.88 (4)	Ni2^xxv^—Dy2—Ni1	132.30 (4)
Ni1^iv^—Dy1—Ni1^xiii^	115.72 (3)	Ni2^ii^—Dy2—Ni1	47.70 (4)
Ni3^ii^—Dy1—Ni1^xiii^	49.26 (2)	Ni2^xi^—Dy2—Ni1	132.61 (4)
Ni3^xi^—Dy1—Ni1^xiii^	91.38 (3)	Ni2^xxvi^—Dy2—Ni1	89.97 (4)
Ni3^xii^—Dy1—Ni1^xiii^	91.39 (3)	Ni2^xii^—Dy2—Ni1	90.03 (4)
Ni1^i^—Dy1—Ni1^xiii^	47.32 (4)	Ni2^i^—Dy2—Ni1^xxvii^	132.61 (4)
Ni1^viii^—Dy1—Ni1^xiv^	94.88 (4)	Ni2^xxv^—Dy2—Ni1^xxvii^	47.70 (4)
Ni1^ix^—Dy1—Ni1^xiv^	94.88 (4)	Ni2^ii^—Dy2—Ni1^xxvii^	132.30 (4)
Ni1^x^—Dy1—Ni1^xiv^	115.72 (3)	Ni2^xi^—Dy2—Ni1^xxvii^	47.39 (4)
Ni1—Dy1—Ni1^xiv^	141.67 (2)	Ni2^xxvi^—Dy2—Ni1^xxvii^	90.03 (4)
Ni1^vi^—Dy1—Ni1^xiv^	115.72 (3)	Ni2^xii^—Dy2—Ni1^xxvii^	89.97 (4)
Ni1^iv^—Dy1—Ni1^xiv^	141.67 (2)	Ni1—Dy2—Ni1^xxvii^	180.00 (3)
Ni3^ii^—Dy1—Ni1^xiv^	91.38 (3)	Ni2^i^—Dy2—Ni1^vi^	89.97 (4)
Ni3^xi^—Dy1—Ni1^xiv^	49.26 (2)	Ni2^xxv^—Dy2—Ni1^vi^	132.30 (4)
Ni3^xii^—Dy1—Ni1^xiv^	91.39 (3)	Ni2^ii^—Dy2—Ni1^vi^	47.70 (4)
Ni1^i^—Dy1—Ni1^xiv^	47.32 (4)	Ni2^xi^—Dy2—Ni1^vi^	90.03 (4)
Ni1^xiii^—Dy1—Ni1^xiv^	47.32 (4)	Ni2^xxvi^—Dy2—Ni1^vi^	47.39 (4)
Ni1^i^—Ni2—Ni1^xiv^	60.75 (7)	Ni2^xii^—Dy2—Ni1^vi^	132.61 (4)
Ni1^i^—Ni2—Ni1^xiii^	60.75 (7)	Ni1—Dy2—Ni1^vi^	45.99 (4)
Ni1^xiv^—Ni2—Ni1^xiii^	60.75 (7)	Ni1^xxvii^—Dy2—Ni1^vi^	134.01 (4)
Ni1^i^—Ni2—Ni1^xv^	108.61 (4)	Ni2^i^—Dy2—Ni1^iv^	47.39 (4)
Ni1^xiv^—Ni2—Ni1^xv^	146.078 (19)	Ni2^xxv^—Dy2—Ni1^iv^	89.97 (4)
Ni1^xiii^—Ni2—Ni1^xv^	146.078 (19)	Ni2^ii^—Dy2—Ni1^iv^	90.03 (4)
Ni1^i^—Ni2—Ni1^xvi^	146.078 (19)	Ni2^xi^—Dy2—Ni1^iv^	132.61 (4)
Ni1^xiv^—Ni2—Ni1^xvi^	146.077 (19)	Ni2^xxvi^—Dy2—Ni1^iv^	132.30 (4)
Ni1^xiii^—Ni2—Ni1^xvi^	108.61 (4)	Ni2^xii^—Dy2—Ni1^iv^	47.70 (4)
Ni1^xv^—Ni2—Ni1^xvi^	60.65 (7)	Ni1—Dy2—Ni1^iv^	45.77 (4)
Ni1^i^—Ni2—Ni1^xvii^	146.078 (19)	Ni1^xxvii^—Dy2—Ni1^iv^	134.23 (4)
Ni1^xiv^—Ni2—Ni1^xvii^	108.61 (4)	Ni1^vi^—Dy2—Ni1^iv^	84.91 (3)
Ni1^xiii^—Ni2—Ni1^xvii^	146.077 (19)	Ni2^i^—Dy2—Ni1^xxviii^	90.03 (4)
Ni1^xv^—Ni2—Ni1^xvii^	60.65 (7)	Ni2^xxv^—Dy2—Ni1^xxviii^	47.70 (4)
Ni1^xvi^—Ni2—Ni1^xvii^	60.65 (7)	Ni2^ii^—Dy2—Ni1^xxviii^	132.30 (4)
Ni1^i^—Ni2—Dy2^xviii^	73.14 (3)	Ni2^xi^—Dy2—Ni1^xxviii^	89.97 (4)
Ni1^xiv^—Ni2—Dy2^xviii^	125.86 (8)	Ni2^xxvi^—Dy2—Ni1^xxviii^	132.61 (4)
Ni1^xiii^—Ni2—Dy2^xviii^	73.14 (3)	Ni2^xii^—Dy2—Ni1^xxviii^	47.39 (4)
Ni1^xv^—Ni2—Dy2^xviii^	72.94 (3)	Ni1—Dy2—Ni1^xxviii^	134.01 (4)
Ni1^xvi^—Ni2—Dy2^xviii^	72.94 (3)	Ni1^xxvii^—Dy2—Ni1^xxviii^	45.99 (4)
Ni1^xvii^—Ni2—Dy2^xviii^	125.53 (8)	Ni1^vi^—Dy2—Ni1^xxviii^	180.00 (5)
Ni1^i^—Ni2—Dy2^xv^	73.14 (3)	Ni1^iv^—Dy2—Ni1^xxviii^	95.09 (3)
Ni1^xiv^—Ni2—Dy2^xv^	73.14 (3)	Ni2^i^—Dy2—Ni1^viii^	132.30 (4)
Ni1^xiii^—Ni2—Dy2^xv^	125.86 (8)	Ni2^xxv^—Dy2—Ni1^viii^	47.39 (4)
Ni1^xv^—Ni2—Dy2^xv^	72.94 (3)	Ni2^ii^—Dy2—Ni1^viii^	132.61 (4)
Ni1^xvi^—Ni2—Dy2^xv^	125.53 (8)	Ni2^xi^—Dy2—Ni1^viii^	47.70 (4)
Ni1^xvii^—Ni2—Dy2^xv^	72.94 (3)	Ni2^xxvi^—Dy2—Ni1^viii^	89.97 (4)
Dy2^xviii^—Ni2—Dy2^xv^	120.000 (1)	Ni2^xii^—Dy2—Ni1^viii^	90.03 (4)
Ni1^i^—Ni2—Ni2^xix^	107.20 (9)	Ni1—Dy2—Ni1^viii^	102.42 (5)
Ni1^xiv^—Ni2—Ni2^xix^	54.54 (7)	Ni1^xxvii^—Dy2—Ni1^viii^	77.58 (5)
Ni1^xiii^—Ni2—Ni2^xix^	107.20 (9)	Ni1^vi^—Dy2—Ni1^viii^	84.91 (3)
Ni1^xv^—Ni2—Ni2^xix^	106.72 (9)	Ni1^iv^—Dy2—Ni1^viii^	84.91 (3)
Ni1^xvi^—Ni2—Ni2^xix^	106.72 (9)	Ni1^xxviii^—Dy2—Ni1^viii^	95.09 (3)

Symmetry codes: (i) −*x*+2/3, −*y*+1/3, −*z*+1/3; (ii) *x*+1/3, *y*+2/3, *z*−1/3; (iii) −*x*+*y*+1, −*x*+1, *z*; (iv) −*y*+1, *x*−*y*, *z*; (v) −*y*+1, *x*−*y*+1, *z*; (vi) −*x*+*y*, −*x*+1, *z*; (vii) *x*+1, *y*+1, *z*; (viii) *x*−1, *y*−1, *z*; (ix) −*y*, *x*−*y*, *z*; (x) −*x*+*y*, −*x*, *z*; (xi) *x*−2/3, *y*−1/3, *z*−1/3; (xii) *x*+1/3, *y*−1/3, *z*−1/3; (xiii) *y*−1/3, −*x*+*y*+1/3, −*z*+1/3; (xiv) *x*−*y*−1/3, *x*−2/3, −*z*+1/3; (xv) *x*−1/3, *y*−2/3, *z*+1/3; (xvi) −*y*+2/3, *x*−*y*+1/3, *z*+1/3; (xvii) −*x*+*y*−1/3, −*x*+1/3, *z*+1/3; (xviii) *x*+2/3, *y*+1/3, *z*+1/3; (xix) −*x*−2/3, −*y*−1/3, −*z*+2/3; (xx) −*x*+1/3, −*y*+2/3, −*z*+2/3; (xxi) *x*−1/3, *y*+1/3, *z*+1/3; (xxii) −*x*+1/3, −*y*−1/3, −*z*+2/3; (xxiii) *x*−*y*+1/3, *x*−1/3, −*z*+2/3; (xxiv) *y*−2/3, −*x*+*y*−1/3, −*z*+2/3; (xxv) −*x*−1/3, −*y*−2/3, −*z*+1/3; (xxvi) −*x*−1/3, −*y*+1/3, −*z*+1/3; (xxvii) −*x*, −*y*, −*z*; (xxviii) *x*−*y*, *x*−1, −*z*.
